# Supplementary material for: Meta-Analysis of Hypoxic Transcriptomes from Public Databases
Source: Biomedicines. 2020 Jan 9;8(1):10. doi: 10.3390/biomedicines8010010 (PMC7168238; doi:10.3390/biomedicines8010010)
Supplement: Supplementary file 1 [file biomedicines-08-00010-s001.pdf]

Supplementary Materials:

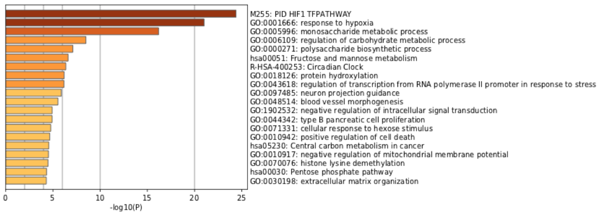

(**A**)

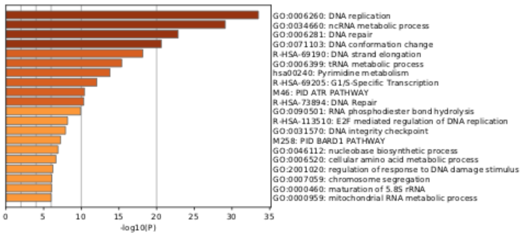

(**B**)

Figure S1: Gene set enrichment analysis of genes up- and downregulated in hypoxic transcriptomes. (A) Gene set enrichment analysis of genes with HN-score => 32; 374 genes (around 1% of all genes). (B) Gene set enrichment analysis of genes with HN-score =< -34; 324 genes (around 1% of all genes)
